# Infective Endocarditis by *Campylobacter* Species—A Narrative Review

**DOI:** 10.3390/pathogens13070594

**Published:** 2024-07-17

**Authors:** Petros Ioannou, Angelos Sourris, Andreas G. Tsantes, George Samonis

**Affiliations:** 1 School of Medicine, University of Crete, 71003 Heraklion, Greece; 2 Department of Internal Medicine and Infectious Diseases, University Hospital of Heraklion, 71110 Heraklion, Greece; 3 Laboratory of Hematology and Blood Bank Unit, “Attikon” University Hospital, School of Medicine, National and Kapodistrian University of Athens, 12462 Athens, Greece; 4 Metropolitan Hospital, Neon Faliron, 18547 Athens, Greece

**Keywords:** *Campylobacter*, infective endocarditis, aortic valve, mitral valve

## Abstract

Infective endocarditis (IE) is a disease that may cause significant morbidity and mortality. IE is classically caused by Gram-positive microorganisms; however, Gram-negative bacteria may seldom also be the cause. *Campylobacter* species cause zoonosis and may also infect humans, mainly causing gastrointestinal infection by *C. jejuni* or invasive disease by *C. fetus*, such as bacteremia, sepsis, meningitis, or vascular infection. *Campylobacter* species IE has rarely been described, and most reports are cases and/or case series. Thus, the characteristics of this disease, including its epidemiology, clinical presentation, treatment, and outcome, remain largely unknown. This study aimed to review all published *Campylobacter* IE cases and describe their characteristics. A thorough search of PubMed, the Cochrane Library, and Scopus for published studies providing information on epidemiology, clinical findings, treatment, and outcome of *Campylobacter* IE cases was performed for the present narrative review. A total of 22 studies containing data from 26 patients were located and included. Among all patients, 73.1% were male; the median age was 65 years. Among all patients, 36.4% had a history of a prosthetic valve. The most commonly affected valve was the aortic, followed by the mitral. Fever, heart failure, and sepsis were the most frequent clinical findings. The most commonly isolated pathogen was *C. fetus*, with only one patient having *C. jejuni* IE. Antimicrobial resistance was low for all antimicrobials, with tetracycline having the highest resistance. Aminoglycosides and beta-lactams were the most commonly used antimicrobials. Surgery was performed in 48% of patients. The mortality rate was 26.9%. Patients who died were more likely to have sepsis, shock, and heart failure and were less likely to have been treated with aminopenicillins; however, no factor was identified in a multivariate logistic regression model as an independent factor for overall mortality.

## 1. Introduction

Infective endocarditis (IE) involves the infection of the endocardium or prosthetic cardiac material such as a cardiovascular implantable electronic device (CIED) or prosthetic cardiac valves [1]. IE can lead to severe complications such as embolic events and metastatic infections and carries a high mortality and morbidity rate [2,3]. In a recent study of patients with IE, mortality in the hospital was 17% [4]. In a different study, the 30-day and one-year mortality rates in these patients were 14% and 30%, respectively [5]. The most typical pathogens causing IE are Gram-positive microorganisms, such as Staphylococci, Enterococci, and Streptococci. These pathogens may be the microbial cause of IE in up to 75% of cases among those with positive cultures, while cases caused by Gram-negative bacteria may seldom be reported [6,7].

*Campylobacter* species are Gram-negative, non-spore-forming, comma-shaped rods [8]. They are microaerophilic; thus, they grow best in atmospheric conditions with 5% to 10% oxygen, and they all grow at 37 °C, even though some grow best in different temperatures, such as *C. jejuni*, which grows best at 42 °C. Species of this genus were initially isolated in 1909 from aborted sheep fetuses and, along with other similar organisms, were considered subspecies of *Vibrio fetus*. However, due to differences in the (G + C) content and fermentation of carbohydrates compared to other members of the genus *Vibrio*, they were appointed to a new genus [8]. More than ten species have been appointed to the genus *Campylobacter*; however, several taxonomic studies have recently suggested that splitting the genus to other ones, such as *Arcobacter*, and appointing some members of the *Campylobacter* genus to other genera would be more appropriate [8,9,10].

Some *Campylobacter* species can cause human disease. These diseases can be either enteric or extraintestinal [8]. The most common species causing intestinal disease is *C. jejuni*, while *C. fetus* most commonly causes extraintestinal disease [8]. Even though they usually grow in the gastrointestinal tract and create intestinal symptoms, their isolation from fecal specimens is problematic due to their relatively slow growth compared to other enteric bacteria; thus, selective growth techniques or filtration methods are needed for their isolation and identification [8,11,12].

Campylobacteriosis is a zoonosis with worldwide distribution. *Campylobacter* spp. can be commonly found in the gastrointestinal tract of cattle, goats, cats, dogs, rodents, and other animals [8,13]. *C. jejuni* can be identified in many different reservoirs, while *C. fetus* ssp. *fetus* has been isolated from sheep, poultry, cattle, swine, and reptiles. Colonization by *Campylobacter* species in animals may occur early in life and usually leads to a lifelong carrier state; however, significant morbidity or mortality may occur [8]. Humans usually contract the disease through contact with animal enteric content that contaminates meat during the slaughtering process [13]. For example, in developed countries, up to 70% of sporadic *Campylobacter* infections are associated with undercooked poultry consumption [14,15,16]. However, transmission could also occur with direct contact with infected animals [17,18].

Notably, the clinical syndromes of *C. jejuni* and *C. fetus* are different. *C. jejuni* can infect normal hosts of all ages, and the infections may occur quite commonly in clusters. On the other hand, *C. fetus* ssp. *fetus* usually acts as an opportunistic pathogen, infecting patients with underlying conditions, even though healthy people could also be infected; clustering is rare [8]. Moreover, *C. jejuni* typically causes a self-limited intestinal infection, may cause diarrhea, and may be isolated in the feces, while *C. fetus* rarely causes diarrhea and causes a life-threatening systematic disease, including bacteremia, vascular infections, meningitis, and abscesses, and may be commonly isolated in the blood [8].

*Campylobacter* species IE has rarely been described, and most reports are made through case series and reports [19]. Thus, the characteristics of this disease, including its epidemiology, clinical presentation, treatment, and outcomes, remain largely unknown. To that end, this study aimed to review all published cases of IE by *Campylobacter* species and describe patients’ characteristics, clinical findings, treatment, and outcomes.

## 2. Materials and Methods

### 2.1. Search Strategy and Inclusion and Exclusion Criteria

For this narrative review, data regarding *Campylobacter* species IE in humans were extracted and collected from the literature. The primary aim was to provide information about the mortality of this infection and the disease’s epidemiology. Moreover, secondary outcomes were to present data on the exact sites of infection, detailed clinical characteristics of the patients, data about the infection’s microbiology, and the treatment provided to patients with IE. For this review, Scopus, PubMed/Medline, and Cochrane Library databases were searched for eligible articles reporting ‘*Campylobacter* AND endocarditis’. The search was supplemented by ‘*vibrio* AND (*fetus* OR *coli*) AND endocarditis)’. The date of the last search was 17 January 2024. Inclusion criteria for this review included (a) studies with original data, such as case reports, case series, and cohort studies providing information at least about the epidemiology and outcomes of *Campylobacter* species IE in humans. Articles in languages other than English were excluded. Letters to the editor, reviews, and systematic reviews that did not provide original information were also excluded. Articles without access to the full text and those referring to animals or with insufficient patient mortality and epidemiology data were also excluded from further analysis. The references of the included articles were also searched to identify other studies that had not been previously identified. Finally, national registries of patients with IE were evaluated to identify patients with IE by *Campylobacter* species.

### 2.2. Data Extraction and Definitions

The following data were extracted from each included study: year of publication, study type, and country; patients’ demographics (age and gender); patients’ relevant medical history (previous cardiac surgery or cardiac valve replacement, time after valve replacement); infection and relevant microbiology (infection site, microorganism identification, complications, and embolic phenomena); treatment provided; surgical management (if performed); and outcomes (i.e., mortality). The association of mortality with index infection was noted by the authors of each study. The diagnosis of IE was confirmed by the present study’s investigators, based on the data provided in each included study by the authors and the modified 2023 Duke-ISCVID criteria if the diagnosis was at least possible (i.e., having at least one major and one minor criterion or at least three minor criteria) or if adequate pathological data justified a diagnosis of IE [20].

### 2.3. Statistical Analysis

Data are presented as numbers (%) for categorical variables and median (interquartile range, IQR) for continuous variables. Continuous variables were compared using the Mann–Whitney U-test for non-normally distributed variables or the t-test for normally distributed variables. All tests were two-tailed, and a *p*-value equal to or lower than 0.05 was considered significant. A univariate linear regression analysis was conducted to identify factors associated with the all-cause mortality of the patients. More specifically, univariate logistic regression was performed to identify any association between gender, age, presence of prosthetic cardiac valve, bad teeth hygiene or recent dental work, history of a previous episode of IE, history of rheumatic heart disease, location of the infection (mitral, aortic, tricuspid, pulmonary, or IE at multiple valves), presence of fever, embolic phenomena, sepsis, heart failure, antimicrobial treatment, and surgical management with all-cause mortality. Statistics were calculated with GraphPad Prism 6.0 (GraphPad Software, Inc., San Diego, CA, USA). A multivariate logistic regression analysis evaluated the effect of factors previously identified in the univariate analysis model associated with all-cause mortality with a *p* < 0.1. Multivariate analysis was performed using SPSS version 23.0 (IBM Corp., Armonk, NY, USA).

## 3. Results

### 3.1. Included Studies’ Characteristics

**Figure 1:**
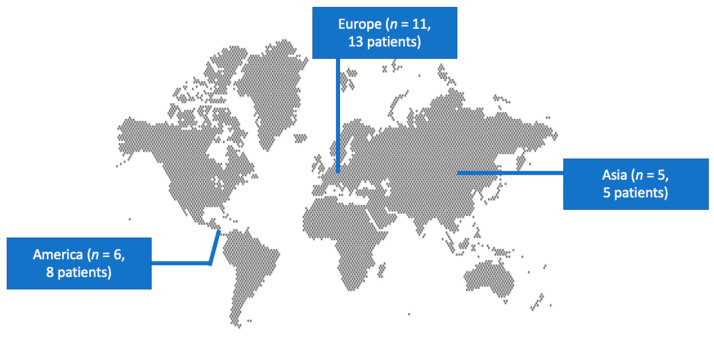
Geographical distribution of *Campylobacter* species infective endocarditis.

A total of 246 articles from Scopus, PubMed/Medline, and Cochrane Library were screened through the initial process. Finally, 22 met the inclusion criteria of the present study and were included [19,21,22,23,24,25,26,27,28,29,30,31,32,33,34,35,36,37,38,39,40,41]. These studies provided data about 26 patients with *Campylobacter* species IE. Table 1 summarizes the characteristics of the included studies. Among them, 12 were conducted in Europe, 7 in North and South America, and 5 in Asia. Figure 1 shows the geographical distribution of *Campylobacter* species IE cases.

### 3.2. Characteristics of Campylobacter Species IE

The median age of patients with *Campylobacter* ΙΕ was 65, ranging from 25 to 95 years, and 73.1% (19 out of 26) were male. History of prosthetic cardiac valve was noted in 36.4% (8 out of 22 patients with available data), congenital heart disease was noted in 22.7% (5), while a recent history of gastroenteritis was present in 21.7% (5 out of 23). Table 1 shows the characteristics of patients with *Campylobacter* species IE in detail.

Blood cultures were positive in 96.2% of patients (25 out of 26 patients). Infection was polymicrobial in 7.7% of patients (2), and the concomitantly isolated pathogens were staphylococci in both patients. The most commonly identified *Campylobacter* species was *C. fetus* in 96.2% of patients (25 out of 26), while *C. jejuni* was identified only in one patient.

Antimicrobial resistance was 13.6% (3 out of 22 strains in studies with available data) to tetracyclines, 9.5% (2 out of 21) to quinolones, 4.5% (1 out of 22) to aminoglycosides, but 0% (0) to aminopenicillins.

Fever was present in only 50% of patients (12 out of 24 with available data), sepsis in 25% (6), shock in 16.7% (4), while heart failure was diagnosed in 29.2% (7), embolic phenomena in 16.7% (4), endophthalmitis in 4.2% (1), and paravalvular abscess in 4.2% (1). Detailed information on the diagnosis and clinical presentation of *Campylobacter* species IE can be seen in Table 2.

### 3.3. Treatment and Outcomes of Campylobacter Species IE

The detailed treatment provided for *Campylobacter* species ΙΕ can be seen in Table 1 and is also summarized in Table 2. Surgical management and antimicrobial therapy were performed in 48% of patients (12 out of 25 patients with available data). All-cause mortality was 26.9% (7 out of 26 patients) and was attributed directly to IE in 19.2% of cases (5).

### 3.4. Statistical Analysis of Campylobacter Species ΙΕ

A statistical comparison of patients with *Campylobacter* species ΙΕ who survived with those who died revealed that those who died were more likely to have presented with sepsis, shock, and heart failure and were less likely to have been treated with aminopenicillins. The statistical comparison results can be seen in Table 1 and Table 2. Moreover, a univariate linear regression analysis of overall mortality with several patients’ characteristics was performed and identified sepsis, shock, and heart failure to be positively associated with overall mortality (*p* < 0.0001, *p* < 0.0001, *p* = 0.0186, respectively). Treatment with aminopenicillin and surgical treatment were negatively associated with mortality (*p* = 0.0186, *p* = 0.362, respectively). A multivariate logistic regression analysis did not identify any factors independently associated with overall mortality.

## 4. Discussion

This study described the characteristics of patients with *Campylobacter* species IE. Most patients were males, recent gastroenteritis was common, most species were *C. fetus*, and the most commonly infected valve was the aortic. The most common clinical findings included fever and heart failure. Aminoglycosides and beta-lactams were the most commonly used antimicrobials, while 27% of patients died.

Even though Gram-positive microorganisms are the most commonly identified pathogens causing IE, Gram-negative microorganisms are also implicated to a smaller extent [6,7]. The Gram-negative pathogens causing IE are traditionally divided into the HACEK group (*Haemophilus* spp., *Aggregatibacter* spp., *Cardiobacterium* spp., *Eikenella* spp., and *Kingella* spp.) and other Gram-negative microorganisms, such as those that are members of the Enterobacterales group, and other non-fermenters [42,43]. This distinction is important due to differences in epidemiology, treatment, and mortality of IE caused by these two groups. Hence, IE by HACEK has a much lower mortality, estimated at 2%. On the other hand, IE by non-HACEK Gram-negative pathogens is more commonly associated with nosocomial acquisition, older patients with more complex medical histories, and quite a higher mortality that can be as high as 40% [43,44,45,46,47,48,49,50]. Notably, there are no uniformly adopted guidelines regarding the treatment of IE by non-HACEK Gram-negative microorganisms. *Campylobacter* IE is rare, even among IE caused by non-HACEK Gram-negative microorganisms. Thus, collecting all published evidence regarding this disease would be essential for providing some advice for physicians treating patients with this rare disease. To that end, this review is the first to provide information regarding the epidemiology, microbiology, clinical characteristics, treatment, and outcomes of *Campylobacter* species IE.

The median age of patients with *Campylobacter* species IE in the present review was 65 years, which is relatively higher than that of patients with IE by non-HACEK Gram-negative bacteria in the literature, which is within the range of 40 to 63 years [48,51,52]. A male predominance was noted, as is also the case in patients with IE by non-HACEK Gram-negative bacteria [48,51,52]. A prosthetic valve was noted in 36.4% of patients with *Campylobacter* species IE, a rate that is within the range of 30% to 59%, which is noted in studies of IE by non-HACEK Gram-negative bacteria [48,51,52]. A cardiac implanted electronic device (CIED) was present in 9.1% of the patients of the present review, while in other studies of patients with IE by non-HACEK Gram-negative bacteria, that rate was as high as 29% [48,51,52]. In another study providing data on patients with IE by *Campylobacter* species, which was not included in the present analysis since it provided aggregated data that could not be used for individual patient analysis, 69.2% occurred in patients with prosthetic valves or intracardiac devices [53]. This high rate resembles that noted in other studies of IE by pathogens associated with foodborne transmission, like *Listeria* or *Salmonella* [54,55]. Notably, in both of these studies, patients were older than those with IE by non-HACEK Gram-negative bacteria, implying that IE by these pathogens that are transmitted through the foodborne route may require more susceptible patients, such as older patients and those with relative immunosuppression [54,55].

A central venous catheter (CVC) was present in 4.5% of patients with *Campylobacter* species IE, a rate lower than in other reports of IE by non-HACEK Gram-negative bacteria, where it ranged from 17% to 20% [48,51,52]. A previous IE episode was noted in the medical history of 9.1% of patients in the present review. In other studies of patients with IE by non-HACEK Gram-negative bacteria, that rate varied from 0 to 67% [48,51,52]. Notably, recent gastroenteritis was mentioned in the recent medical history of patients with *Campylobacter* species IE in 21.7% of cases. This unique characteristic that had not been evaluated in other studies of patients with IE by non-HACEK Gram-negative bacteria is probably associated with the tropism of *Campylobacter* species to the gastrointestinal tract. Importantly, the present review identified *C. fetus* as the almost exclusive cause of IE by *Campylobacter* species, with only one patient having IE by *C. jejuni*. This is of particular importance since it could imply that *C. fetus* may also have the gastrointestinal tract as a portal of entry in these patients, despite the knowledge that *C. fetus* is mainly associated with invasive disease rather than gastrointestinal disease [8].

The most commonly infected valve was the aortic, in 63.6% of patients, and the mitral, in 27.3%. These rates were different in two other studies of IE by non-HACEK Gram-negative bacteria, where the mitral valve was the most commonly infected in 31% of patients, followed by the aortic in 24% in the first study [48], with the aortic being the most commonly infected in 42% of patients, followed by the tricuspid valve in 33% in the second study [52].

Regarding clinical presentation, fever was the most common symptom and was noted in 50% of patients, while 25% were septic. In other studies with IE by non-HACEK Gram-negative bacilli, fever was much more frequently reported in 92% [48,52]. Heart failure was noted in 29.2% of patients with *Campylobacter* species IE, which is similar to the rate seen in other cases of IE by non-HACEK Gram-negative bacteria, that is, from 8% to 37% [48,52]. Embolic phenomena in *Campylobacter* species IE were noted in 16.7%, which is at the lower end of the rate in IE by non-HACEK Gram-negative bacteria, which ranged from 17% to 65% [48,51,52]. A paravalvular abscess was diagnosed in 16% of patients with *Campylobacter* species IE, which is a rate lower than that noted in patients suffering from IE by non-HACEK Gram-negative bacteria where that rate was within the range of 25% to 42% [48,52].

Regarding antimicrobial resistance, all strains in studies with available data were susceptible to aminopenicillins, while resistance to aminoglycosides and quinolones was lower than 10%. Resistance to tetracyclines was 13.6%. In a study evaluating the antimicrobial resistance of 111 strains of *C. fetus* ssp. *fetus* isolated in Canada from 1983 to 2000, similar results to those presented by the present review were seen. Specifically, antimicrobial resistance to aminopenicillins, aminoglycosides, and quinolones was low. However, antimicrobial resistance to tetracyclines was higher than noted in the present review and was 34% [56]. In another relatively older study that also evaluated the antimicrobial resistance of *C. fetus* strains, antimicrobial resistance to aminopenicillins was also zero, while antimicrobial resistance to tetracyclines was 27% [57]. In a more recent study providing data on patients with vascular infections and IE by *Campylobacter* species, tetracycline resistance was high in *C. jejuni* (80%) and was quite low in *C. fetus* (13.2%). Moreover, resistance to ciprofloxacin was 25% in *C. fetus* and 60% in *C. jejuni* [53]. Knowledge about the development of the antimicrobial resistance of *C. fetus* is lacking. At the same time, there is also a lack of epidemiological cut-off values and clinical breakpoints to aid the characterization of strains with antimicrobial resistance [58]. In a recent study, van der Graaf-van Bloois et al. determined the phenotypic susceptibility pattern and the resistome of *C. fetus* by performing whole-genome analysis of 295 *C. fetus* strains [58]. An increase in antimicrobial resistance was noted, and specific differences were observed between *C. fetus* subspecies. Genomic markers for antimicrobial resistance were detected only in *C. fetus* ssp. *fetus* strains and increased after 2000 [58]. Beyond the known intrinsic resistance to nalidixic acid, intrinsic resistance to trimethoprim was noted phenotypically, but a genetic basis was not identified. Resistance genes could be associated with antimicrobial resistance to aminoglycosides and tetracycline. Substitutions in gyrA were defined and conferred resistance to fluoroquinolones. The antimicrobial resistance genes were widely found on mobile elements [58]. Given the abovementioned results of antimicrobial resistance, it is no surprise that aminoglycosides and beta-lactams, such as carbapenems or aminopenicillins, were used to treat *Campylobacter* species IE.

Mortality was notable, with one out of four patients dying, and most succumbed, mainly due to *Campylobacter* species IE. This rate was at the higher end of the range noted in other studies providing data on patients suffering from IE caused by non-HACEK Gram-negative bacteria, where the mortality ranged from 0% to 24% [48,51,52]. In the current study, patients who died were more likely to have had sepsis, shock, and heart failure. At the same time, patients who survived were more likely to have been treated with aminopenicillins. However, no such factor was identified from a multivariate logistic regression analysis as an independent factor for overall mortality. Notably, heart failure and shock have been reported as classical factors associated with mortality in patients with IE in other studies in many different countries, even though these data are derived from patients with IE in general and not by non-HACEK Gram-negative bacteria [59,60,61].

A very recent study presented data from a 5-year retrospective study in France that included 57 patients with bacteremia by *Campylobacter* species [53]. Among these patients, 12 had IE, 44 had vascular infections, and 1 had both. The most frequently identified species was *C. fetus*, similar to the data shown in the present review. However, other species, such as six *C. jejuni*, one *C. lari*, one *C. rectus*, one *C. upsaliensis,* and one *Campylobacter* spp., were also noted. Given that *C. fetus* can cause invasive disease and is by far the most common cause of IE by this species, clinicians may consider performing echocardiography in patients with bacteremia by *C. fetus*, especially when other predisposing factors or clinical signs point towards the diagnosis of IE.

The antimicrobial treatment most commonly included monotherapy with beta-lactams or a combination of beta-lactams with quinolones or aminoglycosides. Mortality was 25%, a rate identical to the one noted in the present review; however, the mortality of patients with IE was 15.4% and was evaluated at three months. Notably, this study had not been included in the current review since the data were aggregated and no data could be extracted to allow individual patient analysis, as performed in the present review.

An important aspect of this study concerns the changes in the diagnosis and treatment of IE over the years. This is particularly important given that the studies that contributed data in the present review date back to 1966, while some were published a few years ago. The diagnosis of IE has changed over the years. IE was first documented by Lazare Riviere in 1646 [62,63]. At the autopsy of a patient who presented with palpitations and irregular pulse and died soon after presentation, “round carbuncles” were noted in the left ventricle, resembling a “cluster of hazelnuts” reaching the “opening of the aorta”. This report has been considered the first reporting aortic valve disease with vegetations of endocarditis [63]. The first detailed description of IE was performed in 1825 and is noted in ‘’Traité Clinique des maladies du Coeur’’ by Jean Baptiste Bouillaud [64]. In that work, the stages of IE are noted, starting from edema to inflammatory and fibrotic changes, and, eventually, calcification and ossification, leading to valvular dysfunction. Emanuel Fredrik Hagbarth Winge and Theodor Albrecht Edwin Klebs, two students of Virchow, suggested that IE was caused by microorganisms, while William Osler recommended a simplification of the complex classifications that were previously used and pointed to the use of the Gram-stain that had been recently introduced to aid towards the diagnosis of IE [63]. Moreover, he described the typical painful red cutaneous nodules seen in IE (Osler nodules). Later in the 1970s, studies reporting on the need to disclose the presence of vegetation in M-mode echocardiography were published [65]. At that time, Stewart et al., from Duke University, reviewed the state of the art and summarized the positive echocardiographic signs but stated that even though echocardiography is a good method to evaluate the presence of IE, routine use may not be encouraged since half of patients with the clinical criteria may have an observable vegetation on echocardiography, while, in patients with a history of IE, echocardiography may have a low diagnostic power due to the slow regression of the vegetation [66]. Even though the presence of fever, anemia, and a murmur led to the suspicion of IE, in 1981, von Reyn performed an analysis of patients with IE with strict definitions and underlined the usefulness of strict definitions in managing suspect IE cases and their need in comparing clinical studies [67]. Later in the 1990s, Duke University made another important contribution by providing a systemization of clinical, microbiological, and echocardiographic characteristics towards the diagnosis of IE [68]. These criteria were later modified in 2000 by Li et al., who provided data from more than 800 patients, and these criteria were largely used in clinical studies later [69]. For the first time, Habib et al. introduce some modifications to the Duke criteria based on novel imaging techniques such as positron emission tomography (PET) [70]. In 2021, the International Society for Cardiovascular Infectious Diseases (ISCVIDs) created a working group of 25 experts from five continents and six subspecialties implicated in the management of IE (infectious diseases, clinical microbiology, cardiovascular pathology, cardiovascular surgery, radiology, and cardiology) to update the diagnostic criteria for IE. This led to the publication of the 2023 Duke-ISCVID IE criteria that proposed important changes in terms of new diagnostic methods in microbiology, intraoperative inspection as a major criterion, new imaging techniques, predisposing conditions, and changes in microbiology to the previous criteria [20]. Given that the present review includes studies presenting cases of IE spanning many different decades, with some of them being published in an era when even echocardiography was not readily available, the means for establishing a diagnosis and the diagnostic accuracy of the criteria used may have differed from study to study. In all the eventually included studies, the investigators assessed all the provided evidence to confirm the diagnosis of IE based on currently applying the 2023 Duke-ISCVID criteria.

The treatment of IE has also undergone substantive changes through the decades. As an example, aminoglycosides were once considered necessary in the treatment of IE in conjunction with another antimicrobial; however, their benefit in treating IE has recently been challenged, leading to a reduction in their use [71]. This has also been noted in the studies published in the present review, where aminoglycosides were used to a lesser extent in more recent studies. Another significant change includes the treatment route for IE. More specifically, intravenous antimicrobials for the total duration of the treatment of IE had always been considered the mainstay of its management. Recently, the POET trial led to a reconsideration of the treatment in some patients suffering from IE if certain conditions are met [72]. Thus, some heterogeneity in the treatment of IE also existed during the decades when the studies included in the present review were published. However, given that Campylobacter species are Gram-negative pathogens, do not belong to the HACEK group, and are rare microbial causes of IE, no specific guidance existed for treating these pathogens. Thus, the treatment of IE by such pathogens has relied on expert opinion or experience derived from relatively small studies of patients with this infection [43,73].

This review has some notable limitations. First, it mainly includes information from case reports. Thus, the present results should be read cautiously, as the quality of evidence was low. Additionally, the number of the included patients is also low, meaning that overly safe conclusions cannot be derived. However, given the rarity of this infection, no data providing adequate information for individual patient analysis could be drawn from larger studies or national registries of patients with IE. Furthermore, data on IE by non-HACEK Gram-negative bacteria are generally scarce, with *E. coli* and *P. aeruginosa* being the most prevalent microorganisms among this rare microbiological group. As an example, non-HACEK Gram-negative bacilli were the cause of 1.8% of IE in a study with 2761 patients with this infection, with none of them suffering from IE by *Campylobacter* species [48]. Moreover, publication bias may have affected the results presented in the present review. Finally, the significant heterogeneity in the diagnosis and treatment of IE over the years may have affected the diagnostic accuracy of the methods used.

## 5. Conclusions

To conclude, this study presents the epidemiological, clinical, and microbiological characteristics of patients with *Campylobacter* species IE as well as their treatment and outcomes. *C. fetus* was the identified species in almost all cases, but gastrointestinal symptoms were noted before the diagnosis of IE in many patients, even though *C. fetus* is classically primarily associated with extraintestinal disease. Thus, in patients with bacteremia by *C. fetus*, echocardiography could be considered to allow for the exclusion of IE. Antimicrobial resistance was minimal; thus, several antimicrobial options exist for treating this infection. Beta-lactams, commonly in combination with aminoglycosides, were used to treat this infection. As in other IE cases by non-HACEK Gram-negative bacteria, mortality was high.

## Figures and Tables

**Table 1:** Characteristics of the included studies.

Study	Number of Patients	Age (Years)	Gender	Site of Infection *n*	Microbiology of Infection, *n*	Treatment Administered, *n*	Infection Outcomes, *n*
Loeb et al., 1966 [21]	2	49, 67	2 Male	1 AoV 1 NR	2 *C. fetus*	2 Penicillin 2 Aminoglycoside 1 Tetracycline	Deaths overall 1 Deaths due to IE 1
Chung et al., 1966 [22]	1	43	Female	NR	*C. fetus*	Penicillin	Deaths overall 1 Deaths due to IE 1
Yoshioka Lee et al., 1970 [23]	1	59	Female	AoV	*C. fetus*	Penicillin Aminoglycoside Tetracycline	Deaths overall 1 Deaths due to IE 1
Wozniak et al., 1985 [24]	1	39	Male	AoV, MV	*C. fetus*	Penicillin Aminoglycoside Surgery	Deaths overall 0
Pönkä et al., 1988 [25]	1	30	Male	AoV, MV	*C. jejuni*	Aminopenicillin Macrolide Aminoglycoside	Deaths overall 0
Caramelli et al., 1988 [26]	1	47	Male	MV	*C. fetus*	Penicillin Aminoglycoside Surgery	Deaths overall 0
Allerberger et al., 1989 [27]	2	70, 84	1 Male 1 Female	1 AoV 1 NR	2 *C. fetus*	1 Aminopenicillin 1 Quinolone 1 Aminoglycoside 1 Surgery	Deaths overall 1 Deaths due to IE 0
Farrugia et al., 1991 [28]	2	60, 76	2 Female	2 AoV	2 *C. fetus*	1 Penicillin 1 Aminopenicillin 1 Quinolone 2 Aminoglycoside 2 Surgery	Deaths overall 0
Abe et al., 1992 [29]	1	52	Male	AoV	*C. fetus*	Aminopenicillin Quinolone Macrolide Aminoglycoside	Deaths overall 0
Peetermans et al., 1992 [30]	1	56	Male	AoV	*C. fetus*	Macrolide Aminoglycoside	Deaths overall 1 Deaths due to IE 1
Mahe et al., 1999 [31]	1	69	Female	TrV	*C. fetus*	Aminopenicillin Quinolone Aminoglycoside Surgery	Deaths overall 0
Miki et al., 1999 [32]	1	63	Male	TrV	*C. fetus*	Carbapenem Aminoglycoside Surgery	Deaths overall 0
Wallet et al., 2003 [33]	1	41	Female	MV	*C. fetus*	Aminopenicillin Quinolone Aminoglycoside Surgery	Deaths overall 0
Haruyama et al., 2006 [34]	1	42	Male	AoV	*C. fetus*	Aminopenicillin Carbapenem Aminoglycoside	Deaths overall 0
Suy et al., 2007 [35]	1	51	Male	AoV	*C. fetus*	Carbapenem Aminoglycoside	Deaths overall 0
Reid et al., 2008 [36]	1	64	Male	AoV	*C. fetus*	Carbapenem Surgery	Deaths overall 0
Sękowska et al., 2009 [37]	1	55	Male	CIED	*C. fetus*	Quinolone Aminoglycoside Surgery	Deaths overall 0
Petridou et al., 2009 [38]	1	65	Male	AoV	*C. fetus*	Aminopenicillin Cephalosporin Macrolide Aminoglycoside	Deaths overall 0
Durovic et al., 2018 [19]	2	71, 89	1 Male 1 Female	1 TrV 1 MV	2 *C. fetus*	2 Carbapenem 1 Surgery	Deaths overall 1 Deaths due to IE 0
Lynch et al., 2018 [39]	1	43	Male	NR	*C. fetus*	NR	Deaths overall 0
Bak et al., 2019 [40]	1	46	Male	AoV, MV	*C. fetus*	Piperacillin/tazobactam Carbapenem Aminoglycoside Surgery	Deaths overall 1 Deaths due to IE 1
Gaultier et al., 2019 [41]	1	49	Female	CIED	*C. fetus*	Aminopenicillin	Deaths overall 0

AoV: aortic valve; CIED: cardiac implantable electronic device; MV: mitral valve TrV: tricuspid valve.

**Table 2:** Patients’ characteristics and infections’ outcome.

Characteristic	All Patients (*n* = 26) *	Survived (*n* = 19) *	Died (*n* = 7) *	*p*-Value
Age, years, median (IQR)	65 (49.5–75.8)	62.5 (48.8–76.3)	67 (55.3–74.3)	0.7575
Male gender, *n* (%)	19 (73.1)	13 (68.4)	6 (85.7)	0.6288
Predisposing factors				
Prosthetic valve, *n* (%)	8/22 (36.4)	6/17 (35.3)	2/5 (40)	1
Congenital heart disease, *n* (%)	5/22 (22.7)	4/17 (3.5)	1/5 (20)	1
Previously on antibiotics, *n* (%)	5/22 (22.7)	3/17 (17.6)	2/5 (40)	0.5481
Recent gastroenteritis, *n* (%)	5/23 (21.7)	4/17 (23.5)	1/6 (16.7)	1
Bad teeth hygiene or recent dental work, *n* (%)	4/22 (18.2)	4/17 (23.5)	0/5 (0)	0.5352
CIED, *n* (%)	2/22 (9.1)	2/17 (11.8)	0/5 (0)	0.2098
Previous IE, *n* (%)	2/22 (9.1)	2/17 (11.8)	0/5 (0)	1
Rheumatic fever, *n* (%)	1/22 (4.5)	1/17 (5.9)	0/5 (0)	1
Central venous catheter, *n* (%)	1/22 (4.5)	0/17 (0)	1/5 (20)	0.2273
Post cardiac surgery, *n* (%)	0/22 (0)	0/17 (0)	0/5 (0)	NA
IVDU, *n* (%)	0/22 (0)	0/17 (0)	0/5 (0)	NA
Method of diagnosis				
Transthoracic echocardiography, *n* (%)	6/22 (27.3)	5/17 (29.4)	1/5 (20)	1
Transesophageal echocardiography, *n* (%)	6/22 (27.3)	5/17 (29.4)	1/5 (20)	1
Autopsy, *n* (%)	3/22 (13.6)	NA	3/5 (60)	NA
Valve culture, *n* (%)	4/22 (18.2)	4/17 (23.5)	0/5 (0)	0.5352
Valve localization				
Aortic valve, *n* (%)	14/22 (63.6)	10/17 (58.8)	4/5 (80)	0.6130
Mitral valve, *n* (%)	6/22 (27.3)	5/17 (29.4)	1/5 (20)	1
Tricuspid valve, *n* (%)	3/22 (13.6)	2/17 (11.8)	1/5 (20)	1
Multiple valves, *n* (%)	3/22 (13.6)	2/17 (11.8)	1/5 (20)	1
CIED, *n* (%)	2/22 (9.1)	2/17 (11.8)	0/5 (0)	1
Clinical characteristics				
Fever, *n* (%)	17/24 (70.8)	8/18 (44.4)	4/6 (66.7)	0.6404
Sepsis, *n* (%)	12/24 (50)	1/18 (5.6)	5/6 (83.3)	0.0008
Heart failure, *n* (%)	7/24 (29.2)	3/18 (16.7)	4/6 (66.7)	0.0381
Shock, *n* (%)	4/24 (16.7)	0/18 (0)	4/6 (66.7)	0.0014
Embolic phenomena, *n* (%)	4/24 (16.7)	3/18 (16.7)	1/6 (16.7)	1
Paravalvular abscess, *n* (%)	1/24 (4.2)	0/18 (0)	1/6 (16.7)	0.2500
Immunological phenomena, *n* (%)	0/24 (0)	0/18 (0)	0/6 (0)	NA
Treatment				
Aminoglycoside, *n* (%)	19/25 (76)	15/18 (83.3)	4 (57.1)	0.2985
Aminopenicillin, *n* (%)	9/25 (36)	9/18 (50)	0 (0)	0.0267
Penicillin, *n* (%)	7/25 (28)	4/18 (22.2)	3 (42.9)	0.3554
Carbapenem, *n* (%)	7/25 (28)	5/18 (27.8)	2 (28.6)	1
Quinolone, *n* (%)	6/25 (24)	5/18 (27.8)	1 (14.3)	1
Macrolide, *n* (%)	4/25 (16)	3/18 (16.7)	1 (14.3)	1
Tetracycline, *n* (%)	2/25 (8)	1/18 (5.6)	1 (14.3)	0.4900
Cephalosporin, *n* (%)	1/25 (4)	1/18 (5.6)	0 (0)	1
Antipseudomonal penicillin, *n* (%)	1/25 (4)	0/18 (0)	1 (14.3)	0.2800
Surgical management, *n* (%)	12/25 (48)	11/18 (61.1)	1 (14.3)	0.0730
Outcomes				
Deaths due to infection, *n* (%)	7 (26.9)	NA	NA	NA
Deaths overall, *n* (%)	5 (19.2)	NA	NA	NA

CIED: cardiac implanted electronic device; IE: infective endocarditis; IQR: interquartile range; IVDU: intravenous drug use; NA: not applicable; *: data are among the number of patients mentioned above unless otherwise described.

## Data Availability

The data presented in this study are available on request from the corresponding author.
